# Whole-body movement analysis using principal component analysis: What is the internal consistency between outcomes originating from the same movement simultaneously recorded with different measurement devices?

**DOI:** 10.3389/fbioe.2022.1006670

**Published:** 2022-11-22

**Authors:** Steven Van Andel, Maurice Mohr, Andreas Schmidt, Inge Werner, Peter Federolf

**Affiliations:** Department of Sport Science, University of Innsbruck, Innsbruck, Austria

**Keywords:** 3D motion capture, optical motion capture, wearable sensors, inertial measurement units IMU, principal component analysis PCA, principal movements PM, gait, walking

## Abstract

A growing number of studies apply Principal Component Analysis (PCA) on whole-body kinematic data to facilitate an analysis of posture changes in human movement. An unanswered question is, how much the PCA outcomes depend on the chosen measurement device. This study aimed to assess the internal consistency of PCA outcomes from treadmill walking motion capture data simultaneously collected through laboratory-grade optical motion capture and field-suitable inertial-based motion tracking. Data was simultaneously collected using VICON (whole-body plug-in gait marker positions) and Xsens (body segment positions) from 20 participants during 2-min treadmill walking. Using PCA, Principal Movements (PMs) were determined using two commonly used practices: on an individual and a grouped basis. For both, correlation matrices were used to determine internal consistency between outcomes from either measurement system for each PM. Both individual and grouped approach showed excellent internal consistency between outcomes from the two systems among the lower order PMs. For the individual analysis, high correlations were only found along the diagonal of the correlation matrix while the grouped analysis also showed high off-diagonal correlations. These results have important implications for future application of PCA in terms of the independence of the resulting PM data, the way group-differences are expressed in higher-order PMs and the interpretation of movement complexity. Concluding, while PCA-outcomes from the two systems start to deviate in the higher order PMs, excellent internal consistency was found in the lower order PMs which already represent about 98% of the variance in the dataset.

## 1 Introduction

Human movement emerges through the coordination of our vastly complex motor system. It is one of the main problems in biomechanics and motor control research to determine how this complex system, with an abundance of degrees of freedom, is coordinated and controlled (Bernstein, 1967). Traditionally, researchers have taken the approach to determine measures that summarize the workings of this system by looking at single outcome variables like the Center of Mass or Center of Pressure (Quijoux et al., 2020; Mehdizadeh et al., 2021; Richmond et al., 2021). However, this approach has also been criticized as such a low-dimensional variable inherently cannot contain all information available within a complex (multi-dimensional) system (Federolf et al., 2021). As an alternative to this low-dimensional approach, the use of Principal Component Analysis (PCA) has been gaining traction in the study of whole-body movement control (Troje, 2002; Daffertshofer et al., 2004; Federolf et al., 2012; Federolf, 2016).

Using PCA, one decomposes the variance in high-dimensional, complex signals into a set of principal component (PC) vectors, each explaining a portion of the total variance. As input for a PCA on human movement, many recent studies have been using body-segment locations represented by marker positions (Federolf et al., 2012; Ross et al., 2018; Armstrong et al., 2021; Mohr et al., 2021). For example, when collecting movement data using a 39-marker full-body marker set, this results in a 117-dimensional input matrix for the PCA (39 markers with an x, y and z dimension). By determining linear relations within this high-dimensional matrix, PCA determines PC vectors, which are orthogonal to each other. The first PC (i.e., PC1) constitutes the vector that can explain the most variance, followed by PC2, PC3 and so on. In this way, the lower-ranked PCs cover the main movement components, sometimes interpreted as main movement strategies (Federolf et al., 2013b). For instance in bipedal postural control, these have been shown to closely approximate whole-body movements such as the ankle and hip strategy (Federolf et al., 2013b). These PC-vectors and the component of the movement they represent are known as “Principal Movements” (PMs; Federolf, 2016).

In gait, PCA has been frequently applied to decompose whole-body movement patterns into separate PMs (Ó′ Reilly, 2021; Promsri, 2022; Stetter et al., 2020; Zago et al., 2022). For example, in treadmill walking, it is typically seen that only a few PMs are needed to explain most of the variance in the movement (Andel et al., 2022; Malloggi et al., 2021; Ó’Reilly & Federolf, 2021). Federolf et al. (2012) determined that already 84.2% of variance could be explained by PM1, representing anterior-posterior arm and leg movement and only one other PM was required to reach more than 90% explained variance (i.e., PM2; 6.6%, representing knee flexion-extension and vertical body movement).

Applying PCA in human movement analysis holds some considerable advantages: 1) the method allows a non-reductionist approach to biomechanical analysis. Previous studies have criticized traditional biomechanical approaches for their reductionist focus (Federolf et al., 2013a, Federolf et al., 2021); trying to understand phenomena such as sports performance or injury risk by focusing on a limited number of variables. Recently, it has been reasoned that movement can only be fully understood as a whole-body system interacting within a surrounding environment (Pinder et al., 2011; Seifert et al., 2013; Andel et al., 2021; Bolt et al., 2021). PCA is very well suited for this purpose, since it provides a coordinate system for complex movements aligned with the variance created through the movement. In this coordinate system, each coordinate axis/basis vector/PC vector/PM explains a known fraction of the variance in the data (eigenvalue). Thus, the complexity of the system can be resembled as precise as required by the research question. 2) PCA is a data-driven method: no *a priori* decisions have to be taken by the researcher, all available information about the system can enter the analysis, thus reducing the investigator bias and the risk of missing an important variable that would have been essential for understanding an underlying mechanism.

As introduced above, PCA provides a non-biased, holistic approach to human movement analysis. However, to fully benefit from these advantages, it is important to also foster the ecological validity and design experiments as close to the performance situation as possible. Here lies one of the limitations in current applications of PCA. That is, while our ability to collect data in the field has increased drastically in recent years through the development of wearable sensors, these wearables have so far found little application in on-field whole-body motion analysis. As such, it is currently unclear whether the results established using laboratory-grade motion capture technology can be generalized or compared with data collected in the field. It is currently unknown whether PCA outcomes determined using different systems are still comparable. Inherent differences should be expected between outcomes, because of the different numbers of markers or measured segments in the analysis, but to what extend this influences the information within the PMs and the outcomes of the analysis is so far unknown.

This study aims to assess the comparability between Principal Movement outcomes, simultaneously collected from the same movement using two different measurement systems. Thereto, we collect kinematic movement data from the same movement (walking on a treadmill) with two independent measurement systems, then independently perform PCAs on the respective datasets, and finally correlate the resultant time series. If the independently obtained PMs represent the same movement pattern, then they carry the same information and correlation coefficients should be close to 1 (±1) for PMs of the same order (hypothesis H1) and close to 0 otherwise (hypothesis H2). We expect that particularly the lower-order PMs, i.e., the main movement patterns, should show this result, while for higher-order movement patterns, the two PCA systems might start to deviate in how the movement information is represented in the PMs. Therefore, for higher-order PMs, we expect to find increasing deviations from 1 on the diagonal of the correlation matrix and increasing deviations from 0 outside the diagonal (hypothesis H3). Finally, PCAs can be calculated separately for each individual, with the disadvantage that resultant PMs cannot be compared between individual volunteers or–after an appropriate normalization (Federolf, 2016; Gløersen et al., 2018)—a single PCA can be calculated on the normalized and concatenated data of all subjects. The latter approach has the advantage of direct comparability of PMs between different participants, but has the disadvantage that the overall PMs are less precisely aligned with individual movement characteristics. Therefore, we conducted our analysis twice, once by calculating the PCAs separately for each individual (_*individual*_PCA) and once for the whole group (_*group*_PCA). We hypothesize (hypothesis H4) that the predicted deviations for higher-order PMs (according to hypothesis H3) will occur earlier (at lower PM order) in the _*group*_PMs compared to the _*individual*_PMs due to the individual differences in movement strategy (hypothesis H4).

## 2 Methods

### 2.1 Participants

Twenty-three participants (13 females) were recruited from the University of Innsbruck student body to be part of this study (average age ± SD: 25.7 ± 4 years; average height ± SD: 175 ± 9 cm; average leg length ± SD: 92 ± 5 cm). All participants were healthy and free of lower limb injuries for at least 6 months before the measurements. Data from three participants had to be excluded from the analysis due to measurement errors, leading to a final sample of N = 20 participants. The protocol of the study was approved by the institutional ethics board (reference number 40/2020) and all volunteers provided written informed consent prior to participation in the study.

### 2.2 Materials and protocol

Participants were equipped for data collection with two separate motion capture systems. First, participants donned a lycra full-body Xsens Link suit with 17 inertial measurement units recording at 240 Hz, distributed over the body (Xsens Technologies, the Netherlands). Second, participants’ movements were recorded using 10 Vicon Bonita infrared cameras at 250 Hz, from 39 reflective markers positioned on top of the lycra suit according to the full-body plugin gait marker position scheme (Vicon Motion Systems, United Kingdom). The x-axis of the Vicon global coordinate system was aligned with the walking direction on the treadmill.

The measurement protocol started with completing a calibration following manufacturer guidelines (Xsens: N-pose and walk protocol, Vicon Nexus: range of motion protocol). After starting data collection in both systems, the participants clapped their hands together to create a recognizable timepoint in both data streams used for an initial synchronization (a precise synchronization was achieved through cross-correlation, as described later). Then, the participant stepped onto a treadmill, which was moving at 4 km/h. Once the participant settled into a steady gait pattern, data was recorded for 2 min.

#### 2.2.1 Data processing

The analysis was aimed at assessing correlations between the data resulting from the two measurement systems, recorded simultaneously during a single bout of activity. To this end, the following data processing steps were implemented.

Data resulting from the Xsens system was processed using MVN Analyze software (Xsens Technologies, the Netherlands) in the “High Definition” mode and “No-level” processing scenario and exported to be further analyzed in Matlab (2019a, the Mathworks, United States). The resulting raw Xsens data presents with the pelvis segment as origin and the x axis in accordance with the orientation of the feet at calibration. To make this consistent across participants and comparable to the VICON system, data was rotated using a custom Matlab algorithm to align the x axis with the direction of walking. Furthermore, Xsens data was resampled from 240 Hz to 250 Hz for consistency between systems. Using the synchronization event that was identifiable in both data streams, data was cut to a portion of 110s of steady state walking to mitigate the effects of stepping on and off the treadmill.

Vicon data were gap-filled using Vicon Nexus software (Vicon Motion Systems, United Kingdom) and exported for further analysis in Matlab. The origin of the data was reset to a position between the two posterior superior iliac spine markers to create a local refence system similar to the pelvis-centered Xsens data. Identification of the synchronization event was used to identify the same 110s of data, which were then exported for further analysis.

As a result of this procedure, for each participant we obtained two independently measured datasets from the same movement, one available as Vicon marker position data and one available as body segment position data exported from XSens. The data processing steps explained in the next paragraphs were executed in parallel on both of these datasets to obtain 2 sets of PMs; ^Vicon^PM_k_ and ^Xsens^PM_k_; where k indicates the order), whose correlation provided a measure of internal consistency of PMs originating from differing measurements.

Further analysis was performed in two common forms of applying PCA to human movement data: an individual as well as a group-level analysis. For both, the PMs were calculated using a Matlab-based software application named *PMAnalyzer* (Haid et al., 2019) following the normalization and analysis steps outlined in the next sections.

#### 2.2.2 Individual-level analysis

Performing separate PCAs for the data of each participant has the advantage that the PC-eigenvectors are optimally aligned with the variance in the specific dataset, i.e., the PMs are optimally aligned with the specific movements of this individual person. A disadvantage is that for every participant a unique coordinate system is created, which means that the PMs are not comparable between individuals, which limits quantitative analyses of differences between individuals or groups.

For the individual-level analysis, each of the 20 participants provided a 27500-by-117 ^Vicon^Data matrix [110 s* 250fps x 39 3D marker coordinates] and a 27,500-by-69 ^Xsens^Data matrix [110 s* 250fps x 23 3D segment position coordinates]. For each participant, both of these matrices were each submitted to a PCA computation to obtain PMkindividualVicon and PMkindividualXsens , which were then correlated to obtain 20 correlation matrices _*individual*_ *R*. Specifically, the first-order PMs, ^Vicon^PM_1_ and ^Xsens^PM_1_ were cross-correlated to obtain best possible time synchronization and the obtained time lag was then applied to all other correlation calculations.

#### 2.2.3 Group-level analysis

After appropriate normalization, data matrices of all participants can be concatenated and submitted together to one common PCA analysis (Federolf, 2016). The advantage of this approach is that the resultant PMs are universal to all participants; consequently, quantitative comparisons of the movement patterns between individuals or between groups of participants become possible. One disadvantage is that these general coordinate axes are now not perfectly aligned with the variance (i.e., the movement structure) within each individual.

Mean Euclidian distances (Federolf et al., 2013b) were used to normalize both data matrices from each individual. Then the data matrices from all volunteers were concatenated to form one single 550000-by-117 input ^Vicon^Data matrix and one single 550000-by-69 input ^Xsens^Data matrix. After calculating the PCA and projecting the data onto the eigenvectors, the resultant scores were split into separate PMs for each participant: PMkgroupVicon and PMkgroupXsens . Correlating these PMs, using the same time lags as in the individual-level analysis, yielded the group-level correlation matrices _*group*_ *R*.

#### 2.2.4 Correlation analysis

To assess the hypotheses stated for the current paper, correlation matrices for the first 16 PMs [k = 1...16] were calculated in both analyses. Note, that the “correlation matrices” _*individual*_ *R* and _*group*_ *R* are not symmetrical since they represent correlations of not the same variables, but correlations between corresponding variables in the other PM matrix; for example, position 2,1 in _*individual*_ *R* is the correlation between ^Vicon^PM_1_ and ^Xsens^PM_2_, whereas position 1,2 is the correlation coefficient of ^Vicon^PM_2_ with ^Xsens^PM_1_. Matrices presenting the mean of the absolute values for the individual correlation coefficientmatrices (averaged over the 20 participants) are presented in the results.

## 3 Results

**FIGURE 1:**
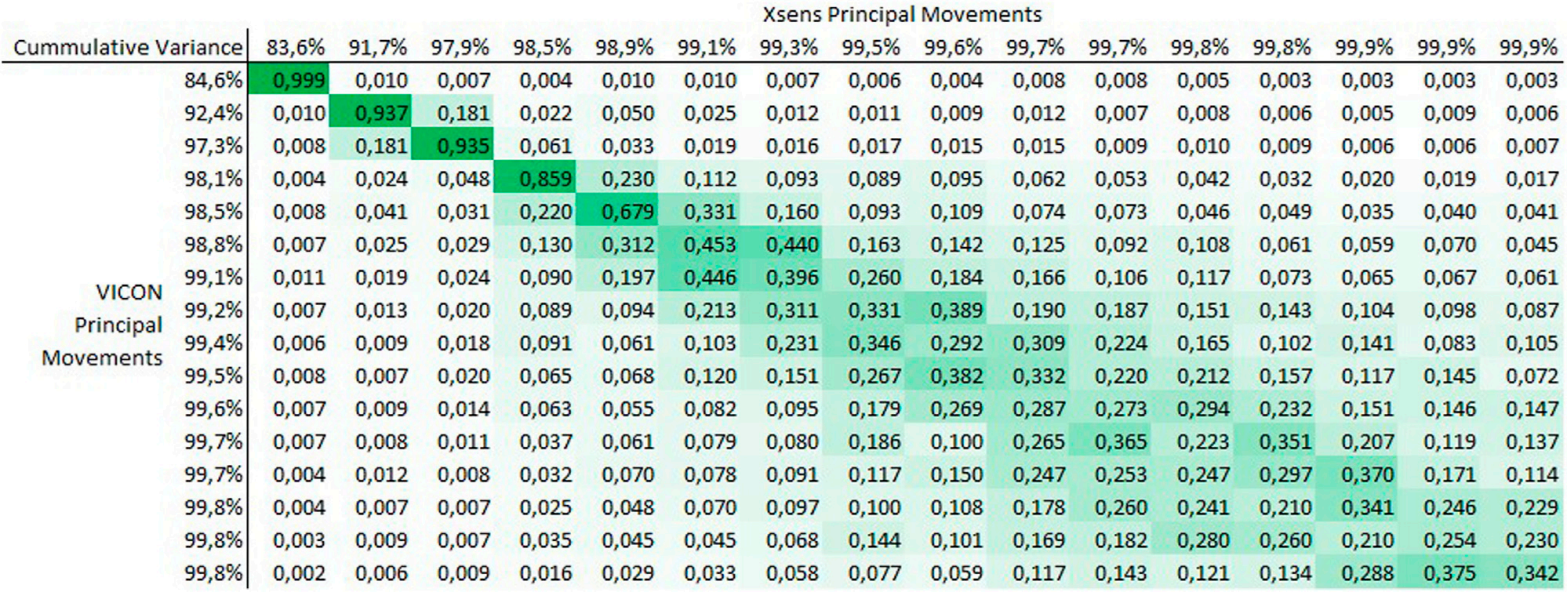
Matrix _individual_R of the correlation coefficients between PMkindividualVicon and PMkindividualXsens . The correlation coefficients shown here represent the mean absolute correlation coefficients averaged over the subject group.

**FIGURE 2:**
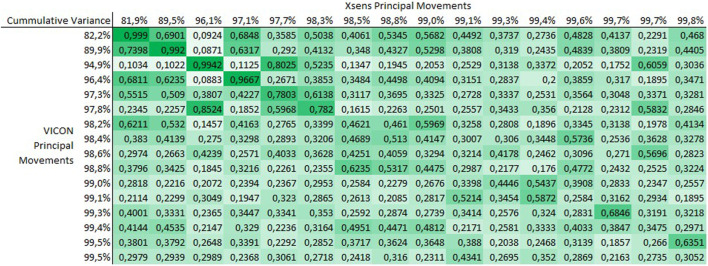
Matrix _group_R of the correlation coefficients between PMkgroupVicon and PMkgroupXsens . The correlation coefficients shown here represent the mean absolute correlation coefficients averaged over the subject group.

Figures 1, 2 show the averaged matrices _*individual*_ *R* and _*group*_ *R*, respectively, where the cell background is colored to provide a visual impression of the correlation results. Figure 1 shows that for the individual analysis, there is a near perfect correlation in the first PC-pair (r greater than 0.999) and still a good agreement in the next four PC-pairs (r greater than 0.67). From the fifth PC-pair on, information appears differently distributed, i.e., the information represented in one PM in the one system is distributed over multiple PMs in the other system.

Figure 2 shows that for the group PCA approach correlations appear more distributed. That is, higher correlations (up to about 0.6) appear more often further away from the diagonal. However, the agreement on the diagonal also appears very good, with the first four PC-pairs correlating greater than 0.95 and the first 6 being correlated greater than 0.78.

## 4 Discussion

The current study investigated internal consistency of PM variables when they are obtained from different measurement systems: a lab-based optical marker tracking system and a wearable IMU system suitable for any environment. Our results demonstrate near perfect internal consistency of the first PM. Correlations of r = 0.999 indicate that PM_1_ derived from either measurement system contain the same information about the participants’ movements. This was true for both approaches to calculating the PCA. Further, for PMs 2 to 4 in the individual-level analysis and for PMs 2 to 6 in the group-level analysis very good internal consistency was observed within PMs of the same order (r > 0.78), confirming hypothesis H1. It should be noted, that these 4 and 6 PMs already represented about 98% of the total movement variance in both PCA types, suggesting that together they provide very close approximations of the analyzed movements.

In the individual-level analysis we also find hypotheses H2 and H3 largely confirmed. Correlation coefficients off the diagonal were close to zero (H2) and for PMs of higher order we find moderate correlation coefficients distributed around the diagonal, suggesting that information represented in one specific PM in one coordinate system is expressed in several PMs in the other coordinate system (H3). Several explanations can be given for H3 and for the observation that the off-diagonal correlation coefficients were not exactly zero. An obvious explanation is that the coordinate systems are not based on the same data in the first place: one data set consisted of reference markers placed on the volunteers’ bodies and one consisted of body segments centers obtained as output of a biomechanical model. Another explanation likely playing a role is that it is impossible to perfectly align the coordinate systems in which the PCA input data were expressed. Particularly the fact that the origins of these coordinate systems are not identical–and probably suffer from relative movements between them–is likely a source for the H3 observation and for the discrepancies to H2.

**FIGURE 3:**
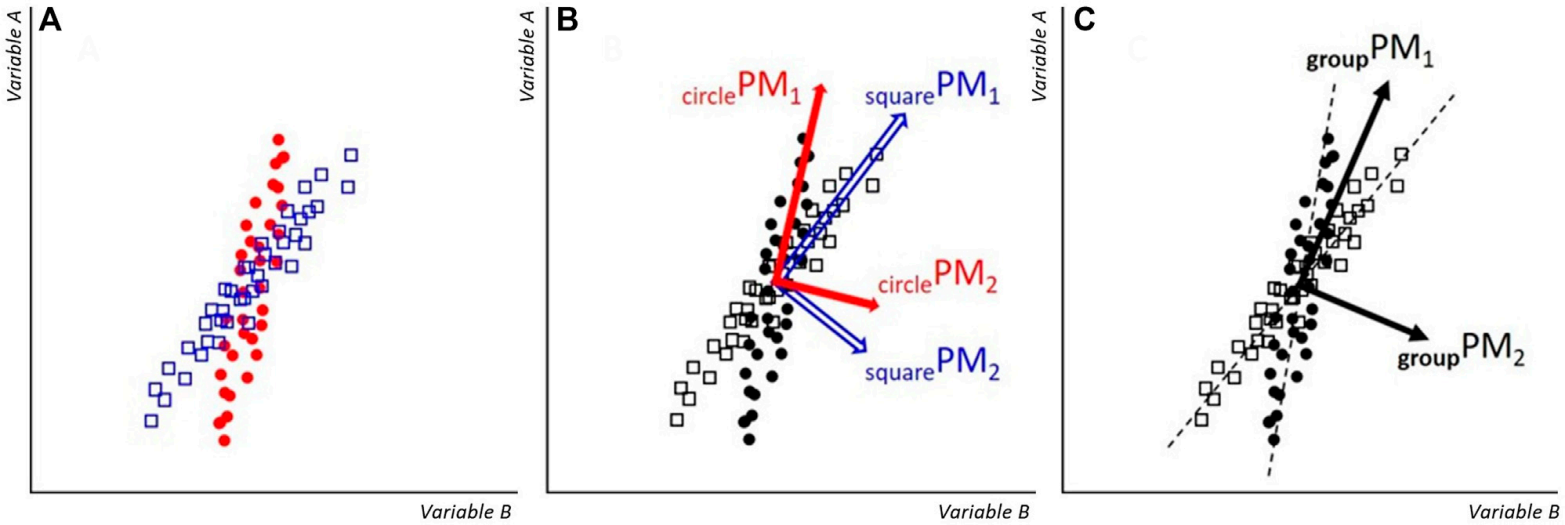
Schematic: **(A)** example datapoints of ‘Variable A’ and ‘Variable B’ from volunteers “square” and “circle”. Since they perform the movement not in exactly the same way, the orientation of the data point clouds are slightly rotated against each other. **(B)** When individual-level PMs are calculated, then each PM-coordinate system aligns with the orientation of the specific data cloud. Thus, the correlation between PM_1_ and PM_2_ will be zero for the data of each volunteer. **(C)** Group-level PMs are calculated for the entire dataset. For each volunteer, _group_PM_1_ is a close approximation of the main movement pattern, however, a small fraction of the volunteer’s main movement pattern also gets projected onto _group_PM_2_. Therefore, if correlations between PM_1_ and PM_2_ are calculated for volunteers separately, non-zero correlation coefficients are obtained, specifically, here a positive correlation results for the squares and a negative correlation for the circles.

The results from the group-level analysis were, at first glance, more surprising. Most notable, for this approach to calculate the PCA, H2 was not confirmed. Moderate and large correlation coefficients were found off the diagonal in all areas of the correlation matrix. We did expect (hypothesis H4) that deviations from zero in the off-diagonal correlations would occur earlier (at lower-order PMs) compared to individual-level analysis, however, we already found such deviations in correlations with _*group*_PM_1_. In a PCA, the resultant eigenvectors are orthogonal (correlations performed over the whole group did in fact result in off-diagonal correlations close to zero, as expected). Therefore, the larger off-diagonal correlation coefficients in _*group*_ *R* have to be a result of separating the concatenated data back into volunteer submatrices after projecting the data onto the _*group*_PMs. The correlations can be explained when considering that different volunteers perform the “same” movement slightly differently, as schematically explained in Figure 3. If this is the main driver behind the larger off-diagonal correlations in the group-analysis, then the same pattern should emerge when computing correlations between PMs of a single measurement system, after separating the concatenated data into separate volunteer-submatrices. Indeed, this prediction was supported by our data (Supplementary Figure S1), corroborating the proposed mechanism.

This property of between-PM correlations in a group-level PCA has important generalizable implications for future applications of PCA. First, when performing a group-level analysis, despite the orthogonality between PC-eigenvectors, independence between PMs of different order cannot be assumed. This is a relevant finding, for example with implications for when evaluating PM data using statistical procedures where independence is an assumption. Furthermore, if a whole group of participants perform a movement systematically different compared to another group, then it is likely that differences in movement strategy can be observed in a higher-order PM. One good example for such opposite behavior can be found in Mohr et al. (2021), where differences in running between the sexes were analyzed and directly opposite behavior of the sexes was in fact found in PM_8_.

PCA offers the opportunity to assess the complexity of a movement pattern, in terms of the dimensionality of the observed movement. That is, if a movement can be accurately summarized by only one PM (e.g., PM_1_ explains a high share of the variance, for example 90%), the movement can be considered less complex than when PM_1_ to PM_4_ are required to reach the same level of explained variance. The current results offer important considerations for this definition of complexity. When using the individual analysis, the explained variance of the lower order PMs is indeed associated to dimensionality of the data and thus movement complexity. However, in the group-analysis the number of PMs required to reach a certain level of explained variance might relate to the movement complexity, as well as to inter-individual differences (Figure 3). As such, care should be taken when interpreting explained variance results stemming from these separate methods.

In summary, this study aimed to assess the comparability between PCA outcomes determined from two separate measurement systems, from the same movement. To conclude, the results of the suggest good internal consistency in lower-order PMs (in fact, near perfect internal consistency in PM_1_). The study also showed, that particularly in the group-level analysis, correlations can also be found between PMs of different order. This finding has important implications for applications of the PMs, particularly for the sensitivity of higher-order PM variables, PM-based measures of complexity and for the statistical treatment of PM-based results.

## Data Availability

The datasets presented in this study can be found in online repositories. The names of the repository/repositories and accession number(s) can be found below: https://osf.io/rz8qf/.

## Supplementary material

SUPPLEMENTARY FIGURE S1 Matrix _group_R of the correlation coefficients between PMkgroupVicon and PMkgroupVicon . Correlations were determined after separating PCA results back into individual matrices, coefficients shown here represent the mean absolute correlation coefficients averaged over the entire subject group.
