# Supplementary figures and images for: Whole-body movement analysis using principal component analysis: What is the internal consistency between outcomes originating from the same movement simultaneously recorded with different measurement devices?
Source: Front Bioeng Biotechnol. 2022 Nov 22;10:1006670. doi: 10.3389/fbioe.2022.1006670 (PMC9723128; doi:10.3389/fbioe.2022.1006670)

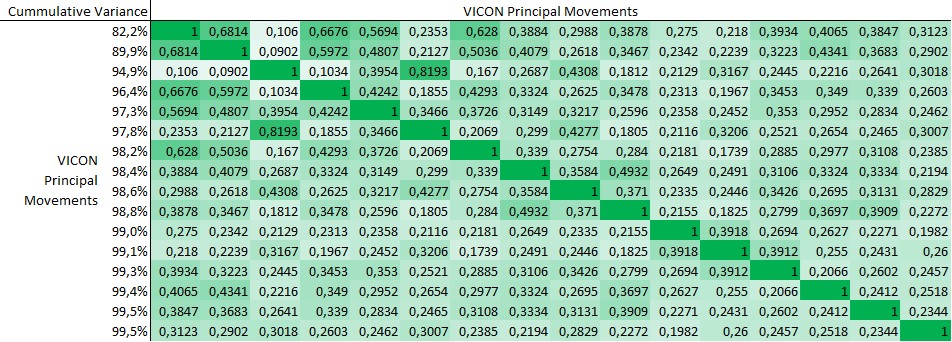

Supplement: Supplementary file 1 [file Figure3.JPEG]
